# Delayed rupture of a right ventricular pseudoaneurysm following transvenous lead extraction

**DOI:** 10.1016/j.hrcr.2026.06.030

**Published:** 2026-06-30

**Authors:** Takuya Nishimura, Mitsuru Wada, Toshihiro Nakamura, Ayumi Ikuta, Naonori Kawamoto, Hiroki Horinouchi, Kengo Kusano

**Affiliations:** 1 Department of Cardiovascular Medicine, National Cerebral and Cardiovascular Center, Suita, Japan; 2 Departments of Cardiac Surgery, National Cerebral and Cardiovascular Center, Suita, Japan; 3 Department of Radiology, National Cerebral and Cardiovascular Center, Suita, Japan

**Keywords:** Complications, Epicardial adipose tissue, Lead extraction, Right ventricular pseudoaneurysm, Cardiac tamponade

**Key Teaching Points**

• Right ventricular pseudoaneurysm is a rare but potentially serious complication of transvenous lead extraction.
• Transmural perforation may occur when epicardial adipose tissue adheres to the lead tip, necessitating surgical repair.
• The clinical presentation of a right ventricular pseudoaneurysm may be atypical, necessitating serial monitoring with transthoracic echocardiography and computed tomography.

## Introduction

Transvenous lead extraction (TLE) is an established therapy for the management of device-related infections and lead malfunction. However, it is associated with significant procedural risks, including vascular injury, cardiac perforation, and pericardial effusion.^1^ Most complications occur acutely, whereas delayed adverse events are uncommon and may be challenging to detect. Ventricular pseudoaneurysm is a rare complication that most commonly develops after myocardial infarction or cardiac surgery, with right ventricular (RV) involvement being particularly uncommon.^2^ In recent years, several case reports have described RV pseudoaneurysm after TLE.^3^^,^^4^ However, to the best of our knowledge, no previous study has clearly demonstrated the temporal progression from pseudoaneurysm formation to impending rupture after TLE using serial multimodality imaging. Herein, this case report documents the longitudinal progression of an RV pseudoaneurysm to impending rupture.

## Case report

**Figure 1:**
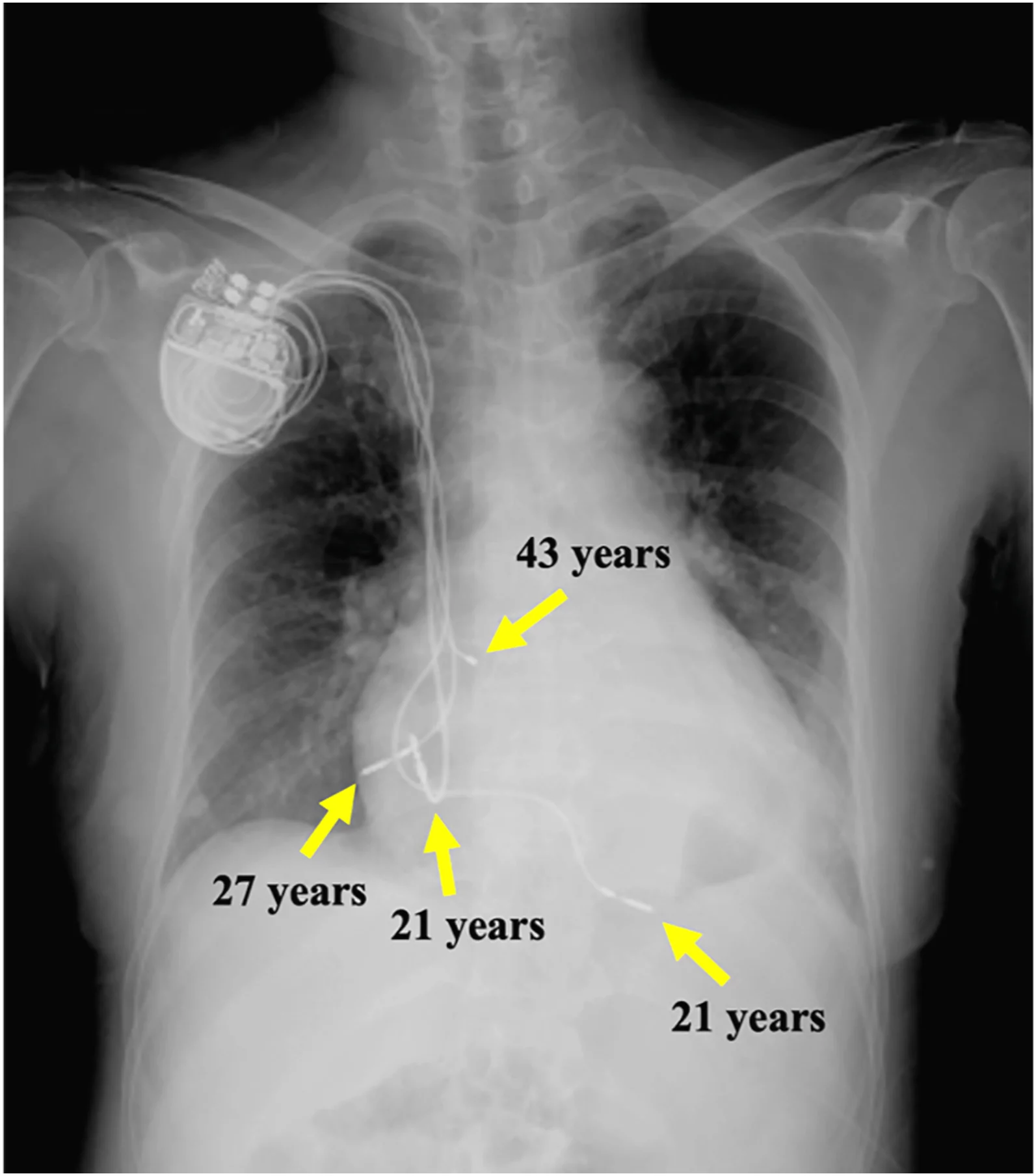
Admission chest radiograph (*anteroposterior seated view*). Right-sided pacemaker system with three leads in the right atrium and one lead in the right ventricle (RV). (*Yellow arrows*) The duration of lead implantation.

This case report describes a 70-year-old woman with a history of sick sinus syndrome (SSS) and permanent pacemaker implantation who was admitted to a local hospital due to a fever for several days. Blood cultures were positive for *Streptococcus dysgalactiae*, and transesophageal echocardiography revealed a right atrial vegetation measuring 19.6 × 8.9 mm, establishing the diagnosis of cardiac device-related infective endocarditis. Subsequently, the patient was transferred to our institution for further management. Upon transfer, intravenous ampicillin–sulbactam therapy was initiated, and preoperative evaluations were performed. Her medications included edoxaban 30 mg daily for paroxysmal atrial fibrillation. The patient had a complex device history. At the age of 25 years, she underwent left-sided VVI pacemaker implantation for SSS. At the age of 27 years, the generator and lead were extracted and replaced with a right-sided AAI pacemaker. The manufacturer and product name of this atrial lead remain unknown. At the age of 43 years, due to increasing atrial capture thresholds, an additional atrial lead (St. Jude Medical 1388T) was implanted. At the age of 48 years, owing to atrial oversensing and progression of SSS, both atrial and ventricular leads (St. Jude Medical 1488T) were added, converting the system to a dual-chamber configuration. At admission, the patient had a right-sided pacemaker generator with 3 leads in the right atrium and 1 lead in the RV (Figure 1). The longest lead dwell time was 43 years, with a total of 4 leads in place. Dense adhesions between the leads and venous structures, extending from the subclavian vein to the superior vena cava, were anticipated. Because the patient had cardiac device-related infective endocarditis requiring complete system removal, the cardiology and cardiothoracic surgery teams jointly reviewed the case and elected to proceed with TLE.

**Figure 2:**
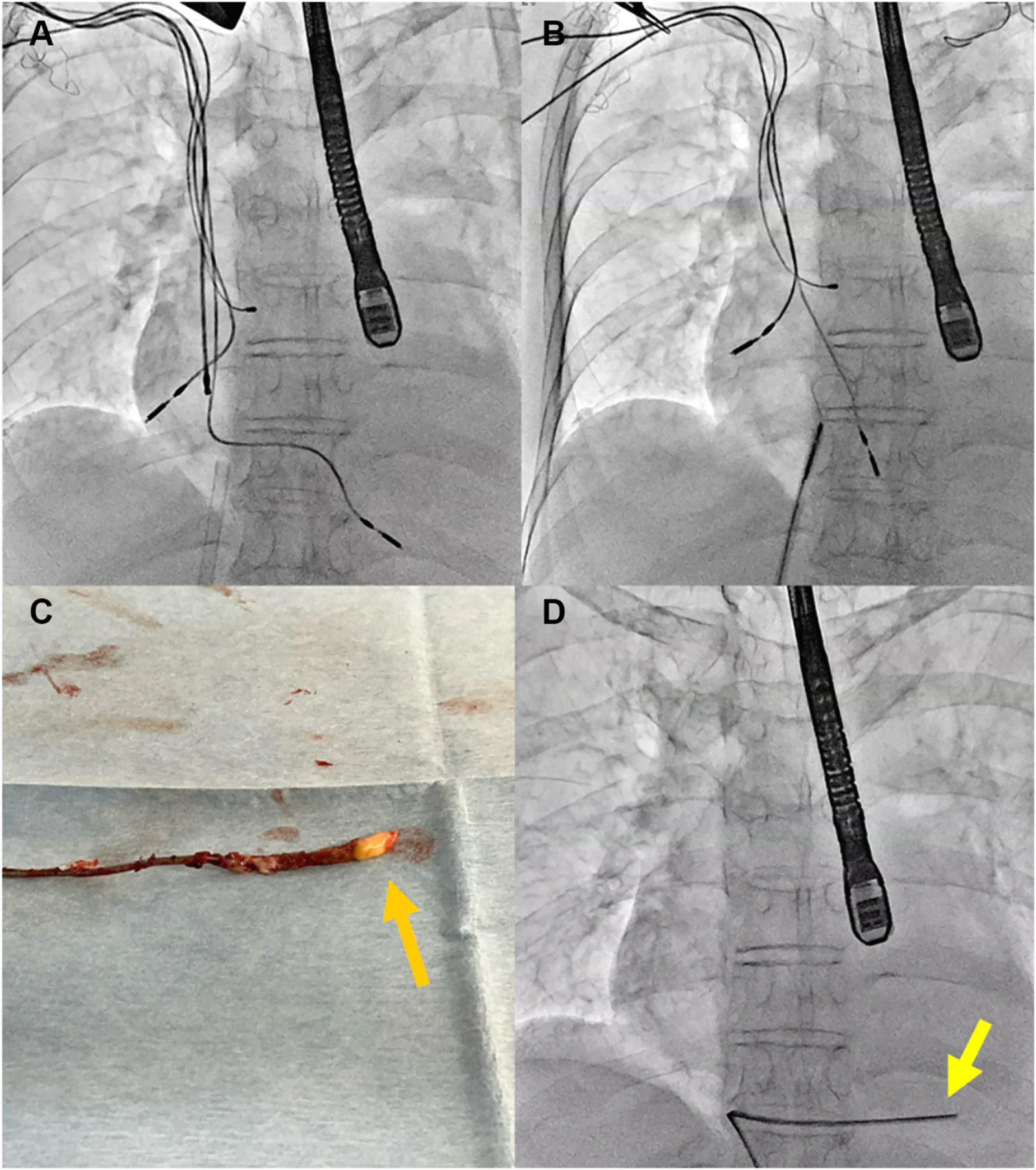
Fluoroscopic images during transvenous lead extraction (TLE) and the extracted RV lead. **A:** Baseline image before TLE. **B:** Detachment of the lead from the RV wall. **C:** The extracted RV lead. Orange arrow indicates adherent epicardial adipose tissue. **D:** Final image following complete TLE. Yellow arrow indicates the pericardial drainage tube. RV = right ventricle.

Initially, the atrial lead with a 27-year dwell time was extracted using an 11-F Evolution RL (Cook Medical, Bloomington, IN). Subsequently, an atrial lead with a 21-year dwell time was removed using a 12-F GlideLight laser sheath (Spectranetics, Philips Company, Colorado Springs, CO). Due to the dense adhesions between the atrial and ventricular leads, the RV lead tip was inadvertently dislodged from the RV wall during atrial lead extraction (Figure 2B). Transesophageal echocardiography revealed a new pericardial effusion consistent with cardiac tamponade, and emergent surgical pericardial drainage was performed. Following drainage, the patient’s vital signs stabilized, and the extraction procedure was resumed. The RV lead was successfully removed using a 14-F GlideLight laser sheath. Myocardial and epicardial adipose tissues adhered to the tip of the extracted RV lead (Figure 2C). Finally, the atrial lead with a 43-year dwell time was extracted using a Needle’s Eye Snare (Cook Medical, Bloomington, IN, USA) and a 14-F GlideLight laser sheath. The pacemaker generator and all 4 leads were successfully removed (Figure 2D). Post-procedure, the patient maintained an intrinsic cardiac rhythm, and temporary pacing was not required.

**Figure 3:**
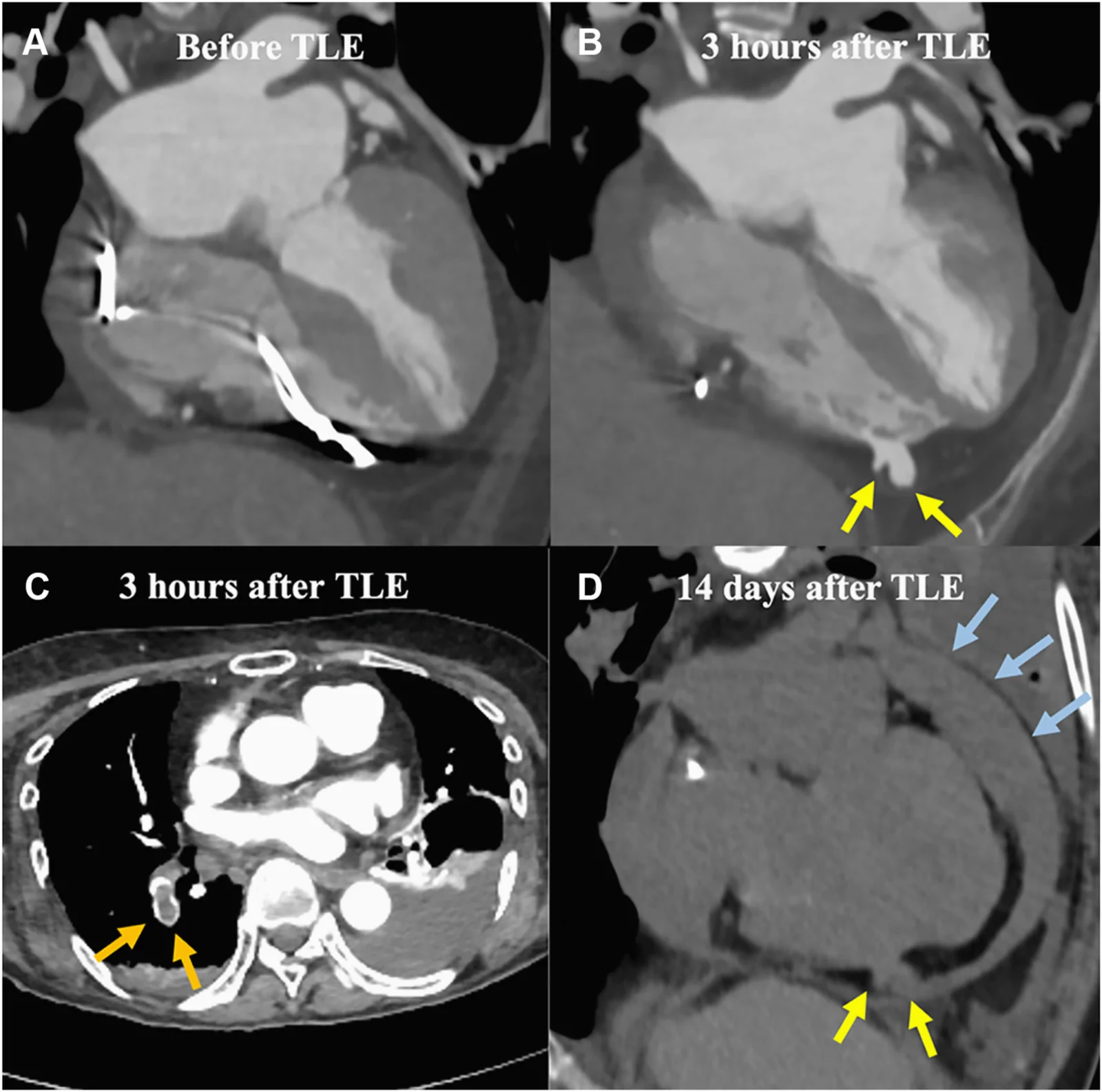
Serial oblique and axial computed tomography images. **A:** On admission (preoperative). **B:** 3 hours after TLE. **C:** An axial computed tomography obtained 3 hours after TLE. **D:** Day 14 after TLE. (*Blue arrows*) Pericardial effusion. (*Yellow arrows*) RV pseudoaneurysm. (*Orange arrows*) Contrast filling defects in branches of the right pulmonary artery. RV = right ventricle; TLE = transvenous lead extraction.

**Figure 4:**
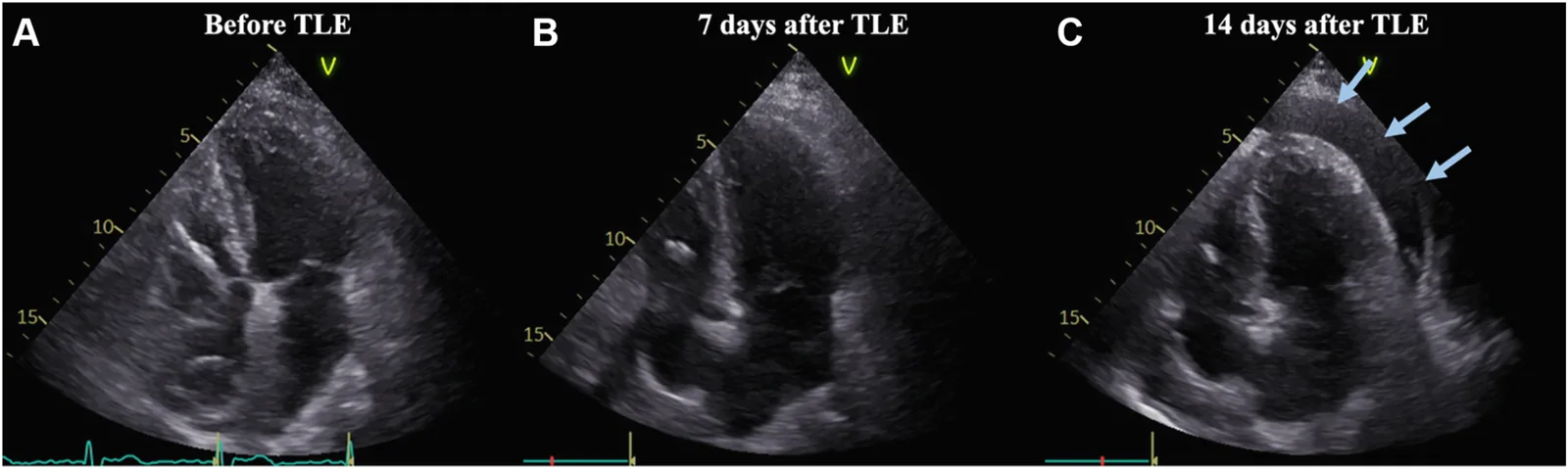
Serial transthoracic echocardiography images. **A:** On admission (preoperative). **B:** 7 days after TLE. **C:** 14 days after TLE. (*Blue arrows*) pericardial effusion. TLE = transvenous lead extraction.

The patient was returned to the ward with a pericardial drain and without catecholamine support. Shortly after arrival, the patient remained hypotensive, with systolic pressures of 60–70 mmHg, necessitating norepinephrine infusion. Contrast-enhanced computed tomography (CT) demonstrated an RV pseudoaneurysm and peripheral filling defects in the branches of the right pulmonary artery, findings that were not present before the TLE (Figure 3A–3C). No significant pericardial effusion was noted on CT, and the drain output did not increase; therefore, the hypotension was considered more likely attributable to intraoperative bleeding or septic pulmonary embolism rather than active bleeding from the RV pseudoaneurysm. Following intravenous norepinephrine administration, her hemodynamics gradually stabilized, and norepinephrine was tapered and discontinued the following day. Edoxaban therapy was resumed on postoperative day (POD) 2, and transthoracic echocardiography (TTE) demonstrated no pericardial effusion. The pericardial drain was removed on POD 4. Rehabilitation was initiated on POD 6; however, on POD 7, the patient developed dyspnea, and chest radiography revealed bilateral pleural effusion. TTE again did not demonstrate clear pericardial effusion, consistent with pre-TLE findings (Figure 4A and 4B). TTE may be insensitive in detecting small or localized pericardial effusions, particularly in regions such as the RV apex. However, as no hemodynamic deterioration suggestive of cardiac tamponade was observed, worsening heart failure was initially suspected, and furosemide treatment was initiated. Despite diuretic treatment, the patient’s clinical response remained inadequate, and dyspnea worsened by POD 14. TTE revealed pericardial effusion encasing both ventricles (Figure 4C). Similarly, non-contrast CT demonstrated circumferential pericardial effusion (Figure 3D). These findings were suggestive of impending rupture of the RV pseudoaneurysm, prompting emergent surgical repair. Intraoperatively, myocardial thinning with perforation was identified at the inferior aspect of the RV apex, and the defect was successfully closed with sutures (Supplemental Video). The patient was subsequently transferred to the intensive care unit, and rehabilitation was continued postoperatively. The patient was ultimately discharged without the need for new device implantation.

## Discussion

RV pseudoaneurysms are exceedingly rare, with few cases documented in the literature.^5^ The clinical presentation is highly variable, ranging from asymptomatic findings to chest pain, dyspnea, and arrhythmia. Reported etiologies include device lead extraction, post-cardiac surgery, penetrating chest trauma, remote myocardial infarction, intracardiac transcatheter interventions, and myocardial biopsy.^2^^,^^3^^,^^6^^,^^7^ Instances of RV rupture attributable to pseudoaneurysms are exceedingly rare. This may reflect either the intrinsic rarity of the condition itself or the relatively lower intracavitary pressures within the RV. Therapeutic strategies for RV pseudoaneurysm include surgical repair, reconstructive patch implantation, percutaneous closure using a septal occlude device, and conservative management; however, no standardized treatment guidelines exist, and management is largely individualized.^8^ Decisions should be guided by the pseudoaneurysm’s anatomical characteristics, the patient’s clinical status, the availability of therapeutic options, and the medical team’s assessment of the risk of rupture.^9^

In the present case report, epicardial adipose tissue adhered to the tip of the extracted RV lead. Previous studies have similarly documented instances in which epicardial fat on the removed lead tip was associated with the subsequent formation of an RV pseudoaneurysm.^3^ This finding may serve as an important intraoperative indicator of transmural myocardial injury and could signify an increased risk of delayed pseudoaneurysm development and rupture. In such patients, early surgical consultation and intensified postprocedural imaging surveillance may be warranted, even when the patient initially appears hemodynamically stable. In retrospect, the preprocedural CT images appeared to show the tip of the RV lead extending beyond the RV endocardium into the epicardial adipose tissue. However, this finding was also considered potentially attributable to imaging artifact, and we have encountered similar preprocedural CT findings in patients who subsequently underwent TLE without complication. Nevertheless, the experience gained from this case highlighted the importance of more proactively considering a hybrid surgical approach in patients with suspicious preprocedural CT findings suggestive of possible myocardial perforation.

In the present patient, hemodynamic stabilization following pericardial drainage allowed the continuation of TLE; however, conversion to an open-chest surgical approach might have been considered at that stage. Although temporary catecholamine support was required after TLE, the pericardial drain did not produce persistent output. Catecholamine support was tapered the following day, the hemodynamics stabilized, and no clinical symptoms were observed. Considering these findings, and given that pseudoaneurysms may remain clinically silent until immediately before rupture, the initial hypotension was more likely attributable to non-rupture factors, such as intraoperative bleeding or septic pulmonary embolism, rather than rupture of the RV pseudoaneurysm. Moreover, postoperative hypotension may have contributed to temporary hemostasis by reducing the intraventricular pressure at the site of injury. However, the subsequent accumulation of pericardial effusion accompanied by worsening dyspnea indicated progression toward impending rupture, necessitating surgical repair. The contributing factors may have included the resumption of edoxaban on POD 2 and the initiation of rehabilitation on POD 6. Although the lower pressure in the right side of the heart may have initially mitigated rapid progression to cardiac tamponade, anticoagulation therapy and increased physical activity associated with expanded activities of daily living likely exacerbated the condition over time.

Although no previous studies have longitudinally documented the imaging course of RV pseudoaneurysms, this case report highlights that such lesions can progress to impending rupture. This temporal pattern underscores the importance of close and repeated imaging surveillance following TLE. Close monitoring is especially recommended if heart failure symptoms emerge after the resumption of antithrombotic therapy or when rehabilitation leads to increased physical activity. Upon detection of recurrent pericardial effusion or pseudoaneurysm enlargement, prompt surgical intervention should be strongly considered.

## Conclusion

RV pseudoaneurysms following TLE may progress silently, culminating in delayed rupture. When intraoperative findings suggest transmural RV injury, serial imaging follow-up is strongly recommended, even if initially stable on imaging.

## Disclosures

Mitsuru Wada received lecture honoraria from Medtronic Japan. Kengo Kusano received lecture honoraria from Medtronic Japan and Daiichi-Sankyo and scholarship funding from Medtronic Japan, Abbott Medical Japan, Boston Scientific Japan, Biotronik Japan, GE Precision Healthcare LLC, Johnson & Johnson, and JSR.
