# Supplementary material for: Delayed rupture of a right ventricular pseudoaneurysm following transvenous lead extraction
Source: HeartRhythm Case Rep. 2026 Jun 30;12(9):1110–4. doi: 10.1016/j.hrcr.2026.06.030 (PMC13593560; doi:10.1016/j.hrcr.2026.06.030)
Supplement: Supplementary file 1 [file mmc1.docx]

**Supplemental Video Legend**

**Intraoperative video of right ventricular pseudoaneurysm repair performed for impending rupture.**

A whitish, markedly thinned perforation site was identified at the right ventricular apex, corresponding to the location of the pseudoaneurysm on computed tomography. Gentle manipulation with forceps resulted in perforation; therefore, the defect was closed with interrupted 4-0 Prolene sutures reinforced with a felt pledget.
